# Automatic seizure detection using three-dimensional CNN based on multi-channel EEG

**DOI:** 10.1186/s12911-018-0693-8

**Published:** 2018-12-07

**Authors:** Xiaoyan Wei, Lin Zhou, Ziyi Chen, Liangjun Zhang, Yi Zhou

**Affiliations:** 1 Department of Biomedical Engineering, Zhongshan School of Medicine, Sun Yat-sen University, Guangzhou, 510080 Guangdong Province China; 2 Software Engineering, School of Computer and Data Science, Sun Yat-sen University, Guangzhou, 510006 Guangdong Province China; 3 Department of Neurology, The First Affiliated Hospital, Sun Yat-sen University, Guangzhou, 510080 Guangdong Province China

**Keywords:** Seizure detection, Epilepsy, Three-dimensional, Convolutional neural network, Multi-channel

## Abstract

**Background:**

Automated seizure detection from clinical EEG data can reduce the diagnosis time and facilitate targeting treatment for epileptic patients. However, current detection approaches mainly rely on limited features manually designed by domain experts, which are inflexible for the detection of a variety of patterns in a large amount of patients’ EEG data. Moreover, conventional machine learning algorithms for seizure detection cannot accommodate multi-channel Electroencephalogram (EEG) data effectively, which contains both temporal and spatial information. Recently, deep learning technology has been widely applied to perform image processing tasks, which could learns useful features from data and process multi-channel data automatically. To provide an effective system for automatic seizure detection, we proposed a new three-dimensional (3D) convolutional neural network (CNN) structure, whose inputs are multi-channel EEG signals.

**Methods:**

EEG data of 13 patients were collected from one center hospital, which has already been inspected by experts. To represent EEG data in CNN, firstly time series of each channel of EEG data was converted into the two-dimensional image. Then all channel images were combined into 3D images according to the mutual correlation intensity between different electrodes. Finally, a CNN was constructed using 3D kernels to predict different stages of EEG data, including inter-ictal, pre-ictal, and ictal stages. The system performance was evaluated and compared with the traditional feature-based classifier and the two-dimensional (2D) deep learning method.

**Results:**

It demonstrated that multi-channel EEG data could provide more information for increasing the specificity and sensitivity in cpmparison result between the single and multi-channel. And the 3D CNN based on multi-channel outperformed the 2D CNN and traditional signal processing methods with an accuracy of more than 90%, an sensitivity of 88.90% and an specificity of 93.78%.

**Conclusions:**

This is the first effort to apply 3D CNN in detecting seizures from EEG. It provides a new way of learning patterns simultaneously from multi-channel EEG signals, and demonstrates that deep neural networks in combination with 3D kernels can establish an effective system for seizure detection.

## Background

An epileptic seizure is a critical clinical problem [1] and Electroencephalogram (EEG) is one of the most prominent ways to study epilepsy and capture changes in electrical brain activities that could indicate an imminent seizure [2]. The diagnosis of epilepsy relies on manual inspection of EEG, which is time-consuming and error-prone. Research from Elger and Hoppe found that only less than half of epileptic seizures which patients document were able to record accurately, and more than half of the seizures captured in long-term video EEG monitoring were not reported [3]. It is of great significance to develop practical and reliable intelligent diagnosis algorithm for automatic seizure detection. Although many efforts have been taken to push the field, we must conclude that seizure detection analysis has not made its way into the clinical practice yet [4].

The task of seizure detection includes distinguishing different stages of seizures, which are generally divided into inter-ictal, pre-ictal and ictal periods [5]. In general, the seizure detection procedure is separated into two parts: feature extraction and classification. There are numerous technological researches based on artificial features and machine learning classifiers [6]. On the one hand, the time-frequency analysis [7], nonlinear dynamics [8], complexity, synchronization [9] and increments of accumulated energy [10] methods were used as feature extraction method. On the other hand, the machine learning classifier includes a Bayes network, traditional neural network and support vector machine (SVM) etc. In fact, feature-classifier engineering techniques have been used successfully in seizure detection tasks [11]. However, the features were extracted based on a limited and pre-fined set of hand-engineer operations. Most importantly, given that seizure characteristics vary among different patients and may change over time, automatically extracting and learning informative features from EEG data is necessary.

Recent advances in deep learning in the past decade have attracted more attention in detective and predictive data analytics, especially in health care and medical practice [12, 13]. It is a powerful computational tool that enables features to be automatically learned from data. Previous studies have proven the deep multi-layer perceptron neural network performs better than the traditional methods such as logistic regression [14] and support vector machine [15]. Related research has shown a 13-layer deep Convolutional neural network(CNN) algorithm achieved an accuracy, specificity, and sensitivity of 88.67, 90.00, and 95.00% respectively in the small Bonn University public data [16]. The ensemble of pyramidal one-dimensional CNN models [17] was proposed to reduce memory space and detection time. Recurrent convolutional neural network learned the general spatially invariant representation of a seizure, exceeding significantly previous results obtained on cross-patient classifiers [18]. The deep unsupervised neural network such as denoising sparse auto-encoder (DSAE) was used in automatically detecting the seizures timely, but may miss important information because of sparse strategy [19]. Other technologies such as deep belief network, transfer learning and so on are also applied to seizure detection [20, 21]. These algorithms based on deep learning lay the foundation of seizure detection research [22].

Nevertheless, the deep neural network was well suited for time series classification [23, 24], it is difficult to learn the corresponding information of multiple electrodes simultaneously. One of the multi-channel analysis is to study different electrodes respectively and finally integrate them [25]. Another method is used by two-dimensional (2D) CNN to learn multi-electrodes, neglecting the relationship between the electrodes [26]. Therefore, we present CNN for seizure detection with a three-dimensional (3D) kernel that is accurate and fully automated to an individual’s need. This method was originally designed to solve the problem of ignoring the inter-frame information recognition of image sequences in the 2D CNN.

In this study, the time series of each channel of EEG data are transformed into images. All channel images consequently were combined as 3D images. In addition, the CNN based on 3D kernels was constructed to perform the classification of different epileptic EEG stages of image datasets. The main contributions of this work are as follows:

1. An efficient method was proposed to preprocess raw EEG data into a 3D image form suitable for a CNN, which integrate multi-channel information;
2. This is the first time that the deep CNN with 3D kernels was applied into the epileptic datasets. In addition, we proposed instructive settings to help the CNN perform well in the seizure detection task.
3. The performance of the 3D CNN methodology was validated by test data, compared to both 2D CNN and traditional machine learning techniques that have been previously evaluated in the literature.

## Methods

### Data resource and data preparation

#### Data resource

The data used in this study were collected from epileptic patients in the electroencephalogram room, Department of Neurology, the First Affiliated Hospital of Xinjiang Medical University, 2013~ 2016. The sampling frequency was 500 Hz and the electrodes were located the international 10–20 system. Clinical experts have labeled every seizure. The specific information of epileptic patients was shown in Table 1.

**Table 1:** The details of collected data

ID	Sex	Age	Channels	State	Time	Seizure	IT
1	F	36	22	AS→SS	8 h	14	654 s
2	F	22	22	AS→SS	48 h	12	274 s
3	F	36	22	AS→SS	8 h	14	1386s
4	F	40	22	AS→SS	24 h	6	302 s
5	M	6	22	AS→SS	24 h	21	453 s
6	F	16	22	AS→SS	24 h	7	329 s
7	F	16	22	AS→SS	24 h	8	254 s
8	F	28	22	AS→SS	24 h	5	400 s
9	F	31	22	AS→SS	24 h	9	423 s
10	M	51	22	AS→SS	24 h	30	1064s
11	M	20	22	AS→SS	24 h	19	4072 s
12	M	46	22	AS→SS	24 h	6	208 s
13	F	15	22	AS→SS	24 h	8	137 s

Notes: *AS* Awake stage, *SS* Sleep stage, *IT* Ictal time

Different seizures have different signal characteristics, and the performance of seizure detection is related to the type of epileptic seizure [27]. So in this paper, the patients’ data with complex partial seizures’ were selected. The experimental data included 13 patients, the age ranged from six to 51 years old. One hundred fifty-nine times of seizures were recorded. The average number of seizures per patient was 12.2. The observation time of each patient was 24 h and the total seizure time was 9956 s.

#### Data preparation

**Fig. 1:**
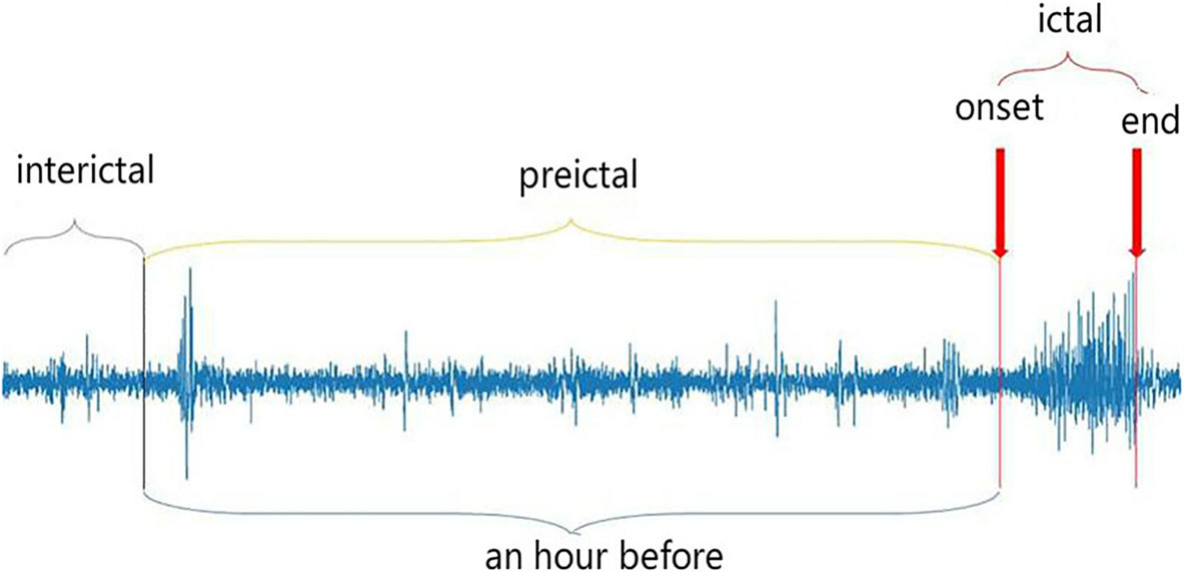
The single-channel EEG recordings illustrating typical brain states. The typical brain states of epilepsy patients include pre-ictal, ictal and inter-ictal three states. An hour segment before each seizure was defined as a pre-ictal state. Neurophysiology experts annotated ictal state. EEG data of the signal that were neither pre-ictal nor ictal defined as inter-ictal states. The figure represents the whole process of brain electrical signal seizure

Numerous investigations have demonstrated a gradual transition between the inter-ictal state and ictal state, which is defined as the pre-ictal stage [28]. Thus, the seizure detection could be considered as the classification of three states. In this study, the EEG data collected from clinical patients were divided into three stages: inter-ictal, pre-ictal and ictal stage, as depicted in Fig. 1. The details are as follows respectively:

- Pre-ictal state: Segment with an hour duration before each seizure was defined as the pre-ictal state [29].
- Ictal state: Neurophysiology experts labeled the clinical seizures.
- Inter-ictal state: The EEG signal data of each patient which was neither pre-ictal nor ictal state were defined as the inter-ictal state.

### System design

**Fig. 2:**
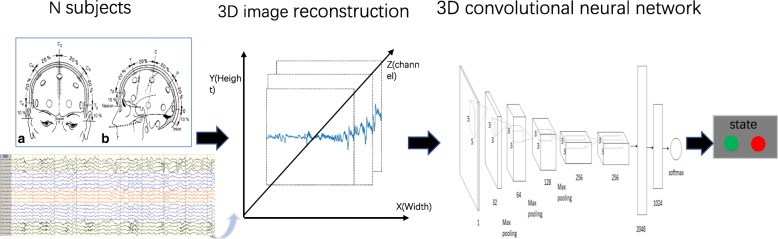
Overview of the pipeline used for seizure detection using 3D CNN

The overall study design consists of typical blocks (see Fig. 2). Firstly, due to the multiple electrodes, the multi-channel EEG time series were constructed as 3D images by means of the position of electrodes on the brain. 3D convolutional kernels were tunable to suit the 3D images input. Moreover, deep CNN automatically learned the patterns of different stages from the EEG signal, and then the training model was used to test in the held-out data. Training and inference phase for 13 patients were calculated using a high-performance computer.

#### Preprocessing

##### Time window selection

A sliding window analysis usually split the raw EEG data into segments for feature extraction, including overlapping sliding window and non-overlapping sliding window [30]. Since EEG signals are non-stationary data, time window should ensure the stability of data. The overlapping sliding window can guarantee the continuity of data, but it is easy to cause information redundancy. Depending on the pre-experiment, the sliding time window for the ictal data is 2500 points (5 s), while for the non-onset period, the sliding time window size is 10 s, and no overlap occurs.

#### 3D image reconstruction

**Fig. 3:**
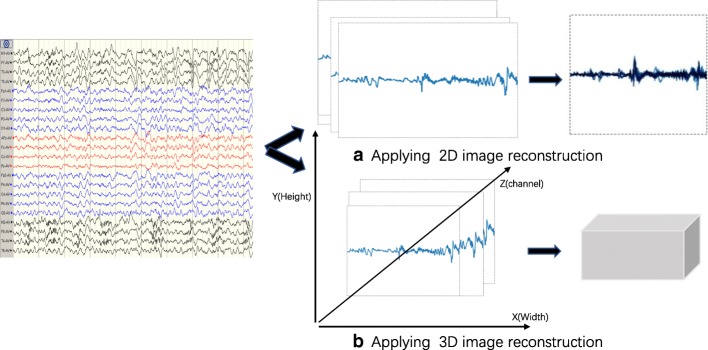
2D and 3D image reconstruction for multi-channel EEG. **a** 2D image reconstruction on a multi-channel time series results in an image in 2D (multiple frames as multiple channels). **b** 3D image reconstruction on multi-channel time series results in 3D image volume, preserving temporal information of the input signal. The z-axis is the channel number, x is the size of the time window, y is the value of the signal

Since a 3D CNN is built in this work, it is inevitable to convert the multi-channel EEG signal into a 3D array (just like the multi-channel image). The conversion must enable to keep most information from the original data. In total, the procedure was divided into two major steps. Firstly, the time series were formed into 2D images. In order to suit the CNN kernels, the image was designed as a square, which resolution is equal to the number of points (like 5000*5000). And the image compression was used to reduce the image down to 256*256 for reducing the complexity of computation. Then the successive relationship of the different electrodes was selected according to the adjacent degree of the electrodes [31], and the corresponding 2D EEG images were fused to form a 3D multi-channel image. Its structure is [256,256, 22], which is presented in the Fig. 3.

#### The proposed 3D CNN structure

The 3D convolution method was proposed in the action recognition in video tasks, which is most widely used as C3D model [32]. Since the CNN based on the 3D kernels has not been used for epileptic classification, there is no optimal network architecture for referring in the literature. Thus, we construct a new CNN structure with the 3D kernel in this experiment, as described in Table 2, which is different from the C3D model and suitable for seizure detection.

**Table 2:** The parameters of the 3D CNN

Layer	Hidden Layer	Related parameters (kernel, kernel size, stride, dropout)		
1	Conv3D + LeakyReLU	64	3*3*3	1*1*1
2	Max Pooling		2*2*2	2*2*2
3	Conv3D + LeakyReLU	128	3*3*3	1*1*1
4	Max Pooling		2*2*2	1*2*2
5	Conv3D + LeakyReLU	256	3*3*3	1*1*1
6	Conv3D + LeakyReLU	256	3*3*3	1*1*1
7	Max Pooling		2*2*2	1*2*2
8	Fully connected	4096		
9	Fully connected	2048		
	Softmax			

##### Feature extraction

**Fig. 4:**
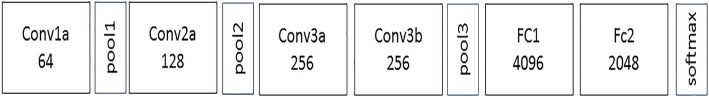
The architecture of 3D CNN. 3D CNN network has 4 convolution layers, 3 max-pooling layers, and 2 fully connected layers, followed by a softmax output layer. All conv3D kernels are 3*3*3 with stride 1 in both three dimensions; all pooling layer kernels are 2*2*2. The first fully connected layer has 4096 output units and the second fully connected layer has 2048 output units

Convolution neural network is a type of neural network with spatial invariance characteristics. In addition, the 3D convolution layer has the ability to collect spatial-temporal information, which preserves the input signal after every convolution operation. We empirically find that 3 × 3 × 3 convolution kernel for all layers to work best among the limited set of explored architectures. The architecture is shown in Fig. 4. As stated in the experiment, the size of the 3D convolution kernel is 3*3*3 and the step length is 1*1*1, with the Leaky Rectified Linear Unit (ReLU) active function whose coefficient is set to 0.01. The pool layer uses the maximum pool and the size of 2*2*2. The step length of the first layer is 2*2*2, and the rest of the layer is 1*2*2, reducing the attenuation of the feature. The third layer is directly connected to the fourth layer to retain the channel characteristic information as far as possible, the full connection layer of 4096 units and 2048 units followed. Finally, the softmax classifier was used for epileptic classification tasks. Our model was implemented in Python 2.7 with Tensor flow 1.6.0.

##### Reduce overfitting stage

For the sake of limited available datasets, it is important to prevent the CNN from overfitting and improve the performance of the model. Firstly, the equal three stage datasets were adopted. Then the dropout strategy was applied in the both of the fully connected layers. Dropout strategy makes results in the dysfunction of the weight of some hidden layer nodes. Thirdly, considering the size of the epoch, group normalization proposed by He [33] have replaced batch normalization algorithm [34] in 3D CNN. Group normalization can divide the data into groups, then calculate the mean and variance in each group. It improves network generalization ability and accelerates the model convergence. The comparison results are shown in Table 3.

**Table 3:** Comparison between batch normalization and group normalization

Method	Batch size = 10		
	Epoch = 1	Epoch = 50	Epoch = 200
BN	74%	84%	**89%**
GN	79%	87%	**90%**

*BN* Batch normalization, *GN* Group normalization. Bold number represents the largest number is that column

##### Classification stage

In this stage, each CNN branch can learn features from different stages. The input of the several branches is the data processed in 3D image reconstruction stage. After the feature extraction stage and reduce overfitting stage, the features obtained by each branch are merged. The outputs of the model are the predicted category labels.

##### Training and inference phase

A total of 36,000 images dataset was split into a training dataset (30,000 images), a validation dataset (3000 images) and a test dataset (3000 images). The training dataset was used to train the parameters of the model. The validation samples used to validate the model. The test dataset was used to evaluate the trained model.

The classification procedure includes the training phase and inference (test) phase. In the training phase, we trained our model using a 10-fold cross-validation strategy. The dataset is randomly scrambled and divided into 10 equal parts. One is selected as the validation dataset to validate the model, and the rest is the training set to complete the training process, each fold data was verified. The aim of this method is to prevent overfitting of the CNN model during training. In the inference phase, the independent test data was used to evaluate the performance of the model.

According to the pre-experiments, we proposed instructive settings to help the CNN perform well with the seizure detection task. The batch size is set as 10, an epoch iteration is 6000 times, and a total of 200 epochs are trained. The cross-entropy loss function is selected as the cost function, using the Adaptive Moment Estimation (Adam) optimizer (initial learning rate = 0.01, β1 = 0.9, β2 = 0.999, decay = 0). For the learning rate strategy: if 10 consecutive Epochs, when the error on the verification set remains unchanged, the current learning rate will be reduced by 10 times. Otherwise, the learning rate is divided by 10 after each 40 Epoch. Repeat the above three operations until training all epochs.

#### Compared 2D CNN structure

**Fig. 5:**
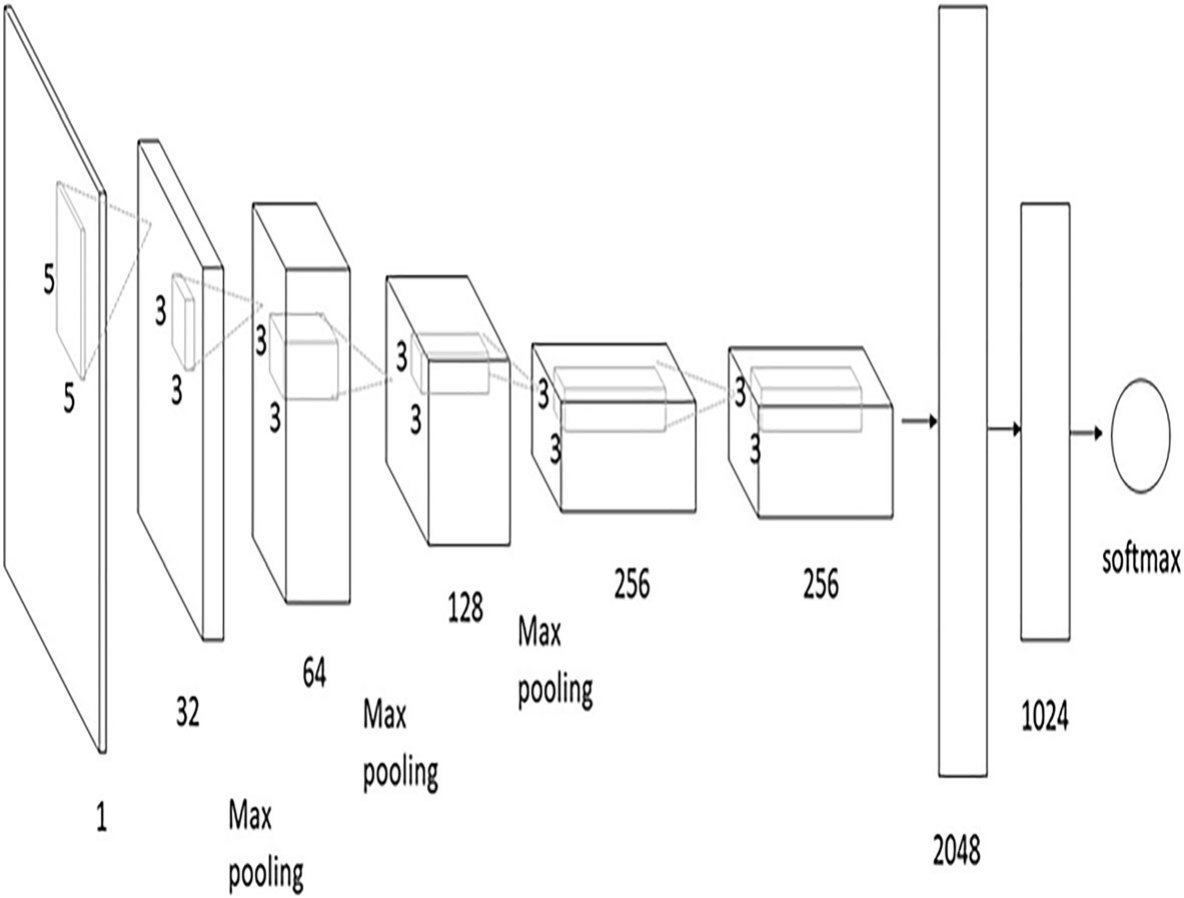
The architecture of 2D CNN. 2D CNN network has 5 convolutions, 5 max-pooling and 2 fully connected layers with a dropout rate of 0.5, followed by a softmax output layer. Conv2D kernels are 3*3 with stride 1or 5*5 with stride1; pooling layer kernels are 2*2 with stride 2 or 3*3 with stride 2. The first fully connected layer has 2048 output units and the second fully connected layer has 1024 output units

The 2D CNN developed rapidly with the help of computer vision, the representative convolution neural network mainly includes LeNet, AlexNet, Inception, ResNet, DenseNet, Xception, MobileNet, ShuffleNet, Capsule network etc. [35]. We constructed the 12 layers 2D CNN structure shown in Table 4 and Fig. 5. For the feature extraction stage, the most difference was 2D convolution layer which was applied to collect EEG image information, and every convolution layer adopted the batch Normalization to reduce the changes in the distribution of internal neurons. For the reduction of overfitting stage, the fully connected layers applied the dropout strategy with a dropout rate of 0.5. For the training phase, the setting including earning rate, epochs and cost function etc. is the same as the 3D CNN.

**Table 4:** The details of 2D CNN structure

Layer	Hidden Layer	Related parameters (kernel, kernel size, stride, dropout)		
1	Conv2D + LeakyReLU+BN	32	5*5	1
2	Max Pooling		3*3	2
3	Conv2D + LeakyReLU+BN	64	3*3	1
4	Max Pooling		2*2	2
5	Conv2D + LeakyReLU+BN	128	3*3	1
6	Max Pooling		2*2	2
7	Conv2D + LeakyReLU+BN	256	3*3	1
8	Max Pooling		2*2	2
9	Conv2D + LeakyReLU+BN	256	3*3	1
10	Max Pooling		2*2	2
11	Fully connected	2048		
	Dropout	0.5		
12	Fully connected	1024		
	Dropout	0.5		
	Softmax

### System evaluation

To evaluate the seizure detection performance, we used the metrics in Table 5 [36].

**Table 5:** Obfuscation matrix of prediction results and actual results

		Prediction		Total
		Object	Non-object	
Actual	Object	True Postive(TP)	False Postive(FP)	TP + FP
	Non-object	False Negtive(FN)	True Negtive(TN)	FN + TN
Total		TP + FN	FP + TN	TP + FP + FN + TN

Each row of the matrix represents the instances in a predicted class while each column represents the instances in an actual class

Standard measurements including sensitivity, specificity, and accuracy were adopted to evaluate the model. According to the above performance parameters, the evaluation indexes are defined as:

1 $$ \mathrm{Accuracy}=\mathrm{TP}+\mathrm{FN}/\mathrm{TP}+\mathrm{TN}+\mathrm{FP}+\mathrm{FN} $$

2 $$ \mathrm{Specificity}=\mathrm{TN}/\mathrm{TN}+\mathrm{FP} $$

3 $$ \mathrm{Sensitivity}\ \left(\mathrm{recall}\ \mathrm{rate}\ \mathrm{or}\ \mathrm{true}\ \mathrm{positive}\right)=\mathrm{TP}/\mathrm{TP}+\mathrm{FN} $$

4 $$ \mathrm{False}\ \mathrm{negative}\ \mathrm{rate}\left(\ \mathrm{FNR}\right)=\mathrm{FN}/\mathrm{TP}+\mathrm{FN}=1-\mathrm{sensitivity} $$

5 $$ \mathrm{False}\ \mathrm{positive}\ \mathrm{rate}\ \left(\mathrm{FPR}\right)=\mathrm{FP}/\mathrm{FP}+\mathrm{TN}=1-\mathrm{specificity} $$

## Results

In this paper, the 2D CNN model was used to test the single- electrode EEG data and multi-electrode EEG data respectively, and the 3D CNN model was tested for demonstrating the 3D kernels’ effeteness compared to other methods. The results are shown in Tables 6, 7 and 8.

**Table 6:** Classification result based on 2DCNN model using single and multi-channel

		Prediction			Accuracy	Specificity	Sensitivity
		Inter-ictal	Pre-ictal	ictal			
Single channel	Inter-ictal	813	124	63	87.53%	90.65%	81.30%
	Pre-ictal	92	864	44	90.20%	92.1%	86.40%
	Ictal	95	34	871	92.13%	94.65%	87.10%
Multi channel	Inter-ictal	822	119	59	88.13%	91.10%	82.20%
	Pre-ictal	107	838	55	89.20%	91.90%	83.80%
	Ictal	71	43	886	92.40%	94.30%	88.60%

**Table 7:** Classification results based on 2D and 3DCNN model using multi-electrode

		Prediction			Accuracy	Specificity	Sensitivity
		Inter-ictal	Pre-ictal	ictal			
3D CNN	Inter-ictal	861	81	58	90.73%	93.05%	86.10%
	Pre-ictal	77	894	29	92.57%	94.15%	89.40%
	Ictal	62	36	902	93.83%	94.15%	90.20%
2D CNN	Inter-ictal	822	119	59	88.13%	91.10%	82.20%
	Pre-ictal	107	838	55	89.20%	91.90%	83.80%
	Ictal	71	43	886	92.40%	94.30%	88.60%

**Table 8:** Performance comparison of different methods

Method	Mean accuracy	Running time
ApEn+DWT + SVM [43]	91.25%	85.1 s
2D CNN	89.91%	3.8 s
3D CNN	**92.37%**	**8.6 s**

*SVM* Support vector machines, *ApEn* Approximate entropy, *DWT* Discrete wavelets transform. The bold number denotes the largest number in that column

According to Table 6, the accuracy rate of the network based on the single electrode data test was 89.95%, the FNR was 15.07%, and the FPR was 7.53%. While the accuracy of the multi-channel was 89.91%, the FNR was 15.13% and the FPR was 7.57%. It demonstrated that more channels from EEG data carried more information and could increase the specificity and sensitivity in medical analysis.

In Table 7, the accuracy of 3D CNN based on multi-channel was 92.37%, the FNR is 11.43%, and the FPR is 6.22%. While the accuracy of the 2D CNN was 89.91%, the FNR was 15.13% and the FPR was 7.57%. The overall recognition rate of the 3DCNN model was higher than that of the 2D CNN, and the recognition rate for the ictal time segment was the highest, followed by the recognition rate of the pre-ictal EEG data.

Table 8 lists the comparison of the 3D CNN based algorithm with traditional machine learning algorithms as well as the 2D CNN, all of the above methods were trained and tested with the data used in this study. According to the results, the method proposed in this paper not only achieved the best performance but also reduce the hand–engineered time.

## Discussion

People with uncontrolled epilepsy suffer uncertainty when a seizure occurs, the diagnosis of seizure was a lack in remote areas because of limited medical services [37]. For examining epilepsy patients efficiently, we hope to develop an automatic seizure detection system to guide doctors.

Deep learning opens the new gate of intelligent diagnosis in medical healthcare, especially in EEG signal processing. The LSTM network was able to predict all 185 seizures, providing high rates of seizure prediction sensitivity based on different pre-ictal time window in the public datasets [38]. The proposed deep learning approach combined the time-frequency and CNN achieves a sensitivity of 81.4, 81.2, and 75% in public dataset [39]. The deep learning applied to the hidden layer makes the expression of data as specific as possible so as to obtain a more efficient representation of EEG signals.

However, most deep learning researches adopt the 2D network, which ignores the fact of multi-channel signal processing [40], Table 6 shows that the more channels EEG signal could improve the performance of the network. We proposed the 3D image reconstruction approach to relate multi-channel information, just like in video processing [41]. In addition, the group normalization, as well as the oversampling techniques were applied to overcome the overfitting of the limited datasets [42]. Compared with the 2D CNN shown in Table 7, our strategy achieved a mean accuracy of more than 90%. It demonstrated that there was a reliable and automatic seizure detection system. This is the first study to introduce 3D kernel CNN’s for seizure detection.

To evaluate our approach, we have measured the proposed algorithm against three studies using the same data, summarized in Table 8. The first method [43] extracted pre-defined features from the EEG data and use conventional machine learning techniques to classify epileptic stages. This requires much time and it is possible that some information is fully or partly missed in the selected features. The next two deep learning method including 2D CNN and 3D CNN have introduced before, which could learn data patterns automatically. On average, the proposed 3D CNN method performs better than 2D CNN in terms of the multi-channel information, and it outperforms the hand-engineered method with less time and high accuracy. A recent competition on Kaggle held the seizure detection contest, the top three winner algorithms [44] includes the hand –engineered and deep learning methods, but they relied on complex features selected. Therefore, the method presented here can be run on an online platform and tested on more data, satisfying the power, resource, and computation that can be implemented in the wearable device.

However, limitations of this work have to be admitted. Firstly, this method, all deep learning technology requires sufficient data to train the model and the design of the network is much harder to guarantee to be optimal. Maybe other research gets better performance just tuning the small parameters. Secondly, few clinical experts in one center labeled the model data. Thirdly, the experiment just involves the EEG data type, which neglects other data types from a multi-scale perspective. In order to have a more generalizable clinical validation, the methods should be tested on an extensive and multi-center dataset. Further relevant information sources can be readily incorporated into the deep neural networks, such as video, weather patterns, biomarkers, or clinical notes [45, 46]. Detection algorithm which incorporates these additional inputs and the data types is the focus of ongoing work.

## Conclusion

This study proposed a new approach for epileptic EEG classification, which constructed the 3D CNN for multi-channel EEG data. The main advantage of the method is fully utilizing the multi-channel signal information without hand-engineered. The 3D CNN model outperformed the previously heuristic detectors. To our best knowledge, this study is the first try of using 3D CNN algorithm for seizure detection. Therefore, it may serve as a benchmark for new work exploring deep learning enabled seizure detection in terms of multi-channel EEG data. Further studies need to carry out to validate this algorithm in the multi-center dataset. We expect more advances in signal processing, network design, model validation to shape the future of automatic seizure detection.

## Abbreviations

2D: Two-dimensional
3D: Three-dimensional
ApEn: Approximate entropy
AS: Awake stage
CNN: Convolutional neural network
DWT: Discrete wavelets transform
EEG: Electroencephalogram
IT: Ictal time
ReLU: Rectified Linear Unit
SS: Sleep stage
SVM: Support vector machines

## References

[1] Jory C, Shankar R, Coker D, Mclean B, Hanna J, Newman C (2016). Safe, and sound? A systematic literature review of seizure detection methods for personal use. Seizure.

[2] Beniczky S, Ryvlin P (2018). Standards for testing and clinical validation of seizure detection devices. Epilepsia.

[3] Elger CE, Hoppe C (2018). Diagnostic challenges in epilepsy: seizure under-reporting and seizure detection. Lancet Neurol.

[4] Mormann F, Andrzejak RG (2016). Seizure prediction: making mileage on the long and winding road. Brain.

[5] Trinka E, Kälviäinen R (2017). 25 years of advances in definition, classification and treatment of status epilepticus. Seizure Eur J Epilepsy.

[6] Cogan D, Birjandtalab J, Nourani M, Harvey J, Nagaraddi V (2016). Multi-biosignal analysis for epileptic seizure monitoring. Int J Neural Syst.

[7] Fiscon G, Weitschek E, Cialini A, Felici G, Bertolazzi P, Salvo SD (2018). Combining EEG signal processing with supervised methods for Alzheimer’s patient's classification. BMC Med Inform Decis Mak.

[8] Zhou Y, Huang R, Chen Z, Chang X, Chen J, Xie L (2012). Application of approximate entropy on dynamic characteristics of epileptic absence seizure. Neural Regen Res.

[9] Gao ZK, Cai Q, Yang YX, Dong N, Zhang SS (2017). Visibility graph from adaptive optimal kernel time-frequency representation for classification of epileptiform EEG. Int J Neural Syst.

[10] Gigola S, Ortiz F, D’Attellis CE, Silva W, Kochen S (2004). Prediction of epileptic seizures using accumulated energy in a multiresolution framework. J Neurosci Methods.

[11] Subasi A, Gursoy MI (2010). EEG signal classification using PCA, ICA, LDA and support vector machines. Expert Syst Appl.

[12] Litjens G, Kooi T, Bejnordi BE, Aaa S, Ciompi F, Ghafoorian M (2017). A survey on deep learning in medical image analysis. Med Image Anal.

[13] Xun G, Jia X, Zhang A (2016). Detecting epileptic seizures with electroencephalogram via a context-learning model. BMC Med Inform Decis Mak.

[14] Subasi A, Erçelebi E (2005). Classification of EEG signals using the neural network and logistic regression. Comput Methods Programs Biomed.

[15] Mirowski PW, Lecun Y, Madhavan D, Kuzniecky R, editors. Comparing SVM and convolutional networks for epileptic seizure prediction from intracranial EEG. Machine Learning for Signal Processing, 2008 MLSP 2008 IEEE Workshop on; 2008.

[16] Acharya UR, Oh SL, Hagiwara Y, Tan JH, Adeli H. Deep convolutional neural network for the automated detection and diagnosis of seizure using EEG signals. Computers in biology and medicine. 2018; 100:270–8. 10.1016/j.compbiomed.2017.09.017 28974302

[17] Ullah I, Hussain M, Qazi EUH, Aboalsamh H (2018). An automated system for epilepsy detection using EEG brain signals based on deep learning approach. Expert Syst Appl.

[18] Thodoroff P, Pineau J, Lim A (2016). Learning robust features using deep learning for automatic seizure detection.

[19] Lin Q, Ye S-q, Huang X-m, Li S-y, Zhang M-z, Xue Y, Chen W-S. Classification of epileptic EEG signals with stacked sparse autoencoder based on deep learning. InInternational Conference on Intelligent Computing: 2016:802–810.

[20] Movahedi F, Coyle JL, Sejdić E (2017). Deep belief networks for electroencephalography: A review of recent contributions and future outlooks. IEEE J Biomed Health Inform.

[21] Jiang Y, Wu D, Deng Z, Qian P, Wang J, Wang G (2017). Seizure Classification from EEG Signals using Transfer Learning, Semi-Supervised Learning and TSK Fuzzy System. IEEE Trans Neural Syst Rehabil Eng.

[22] Zhang Q, Yang LT, Chen Z, Li P (2018). A survey on deep learning for big data. Inf Fusion.

[23] Gupta A, Singh P, Karlekar M (2018). A Novel Signal Modeling Approach for Classification of Seizure and Seizure-free EEG Signals. IEEE Trans Neural Syst Rehabil Eng.

[24] Makinson CD, Tanaka BS, Sorokin JM, Wong JC, Christian CA, Goldin AL (2017). Regulation of Thalamic and Cortical Network Synchrony by Scn8a. Neuron.

[25] Paesschen WV (2018). The future of seizure detection. Lancet Neurol.

[26] Quigg M, Leiner D (2009). Limitations of single-channel EEG on the forehead for neonatal seizure detection. J Perinatol.

[27] Ulate-Campos A, Coughlin F, Gainza-Lein M, Fernández IS, Pearl P, Loddenkemper T. Automated seizure detection systems and their effectiveness for each type of seizure. Seizure. 2016;40:88–101. 10.1016/j.seizure.2016.06.008 27376911

[28] Cook MJ, O'Brien TJ, Berkovic SF, Murphy M, Morokoff A, Fabinyi G (2013). Prediction of seizure likelihood with a long-term, implanted seizure advisory system in patients with drug-resistant epilepsy: a first-in-man study. Lancet Neurol.

[29] Bandarabadi M, Rasekhi J, Teixeira CA, Karami MR, Dourado A (2015). On the proper selection of preictal period for seizure prediction. Epilepsy Behav.

[30] Zhang Z, Chen Z, Zhou Y, Du S, Zhang Y, Mei T (2014). Construction of rules for seizure prediction based on approximate entropy. Clin Neurophysiol.

[31] Weis JA, Miga MI, Yankeelov TE (2016). Three-dimensional image-based mechanical modeling for predicting the response of breast cancer to neoadjuvant therapy. Comput Methods Appl Mech Eng.

[32] Du T, Bourdev L, Fergus R, Torresani L, Paluri M, editors. Learning Spatiotemporal Features with 3D Convolutional Networks. IEEE International Conference on Computer Vision; 2016.

[33] Crytzer TM, Keramati M, Anthony SJ, Cheng Y-T, Robertson RJ, Dicianno BE (2018). Exercise prescription using a group-normalized rating of perceived exertion in adolescents and adults with spina bifida. PM&R.

[34] Ioffe S, Szegedy C (2015). Batch normalization: accelerating deep network training by reducing internal covariate shift.

[35] Alom MZ, Taha TM, Yakopcic C, Westberg S, Hasan M, Essen BCV (2018). The history began from AlexNet: a comprehensive survey on deep learning approaches.

[36] Assi EB, Dang KN, Rihana S, Sawan M (2017). Towards accurate prediction of epileptic seizures: a review. Biomed Signal Process Control.

[37] Ryvlin P, Beniczky S. Seizure detection and mobile health devices in epilepsy: Update and future developments. Epilepsia. 2018;59:7-8. 10.1111/epi.14088 29873830

[38] Tsiouris ΚΜ, Pezoulas VC, Zervakis M, Konitsiotis S, Koutsouris DD, Fotiadis DI. A Long Short-Term Memory deep learning network for the prediction of epileptic seizures using EEG signals. Computers in biology and medicine. 2018;99:24–37. 10.1016/j.compbiomed.2018.05.019 29807250

[39] Truong ND, Nguyen AD, Kuhlmann L, Bonyadi MR, Yang J, Ippolito S, Kavehei O. Convolutional neural networks for seizure prediction using intracranial and scalp electroencephalogram. Neural Networks. 2018;105:104-111. 10.1016/j.neunet.2018.04.018 29793128

[40] Denmark T, Fridrich J, Comesañaalfaro P (2016). Improving selection-channel-aware steganalysis features. Electron Imaging.

[41] Ji S, Xu W, Yang M, Yu K (2012). 3D convolutional neural networks for human action recognition. IEEE Trans Pattern Anal Mach Intell.

[42] Brinkmann BH, Wagenaar J, Abbot D, Adkins P, Bosshard SC, Min C (2016). Crowdsourcing reproducible seizure forecasting in human and canine epilepsy. Brain.

[43] Kumar Y, Dewar ML, Anand RS (2014). Epileptic seizures detection in EEG using DWT-based ApEn and artificial neural network. SIViP.

[44] Baldassano SN, Brinkmann BH, Ung H, Blevins T, Conrad EC, Leyde K (2017). Crowdsourcing seizure detection: algorithm development and validation on human implanted device recordings. Brain A J Neurol.

[45] Kiral-Kornek I, Roy S, Nurse E, Mashford B, Karoly P, Carroll T (2017). Epileptic seizure prediction using big data and deep learning: toward a Mobile system. EBiomedicine.

[46] Schneider JM, Maguire MJ. Identifying the relationship between oscillatory dynamics and event-related responses. International Journal of Psychophysiology. 2018;133:182–192. 10.1016/j.ijpsycho.2018.07.002 29981766

